# Feed Efficiency Classification in Confined Texel Ewe Lambs: Relationships with Ruminal Fermentation, Nitrogen Metabolism, and Greenhouse Gas Emissions

**DOI:** 10.3390/ani16162554

**Published:** 2026-08-16

**Authors:** Charleni Crisóstomo Abdalla, Adibe Luiz Abdalla Filho, Rui José Branquinho de Bessa, Ricardo Lopes Dias da Costa, Letícia de Sousa Corrêa, Josiel Ferreira, Nathalya Sanchez, Vinicius Souza Pestana, Vagner Ovani, Adibe Luiz Abdalla, Helder Louvandini

**Affiliations:** 1 Animal Nutrition Laboratory, Centre for Nuclear Energy in Agriculture, University of São Paulo, Av. Centenário 303, Piracicaba 13416-000, São Paulo, Brazil; nathy.medvet@icloud.com (N.S.); viniciuspestana@fam.edu.br (V.S.P.); vagnerovani@usp.br (V.O.); abdalla@cena.usp.br (A.L.A.); louvandini@cena.usp.br (H.L.); 2 Centro de Investigação Interdisciplinar em Sanidade Animal (CIISA), Faculdade de Medicina Veterinária, Universidade de Lisboa, Av. Universidade Técnica, 1300-477 Lisboa, Portugal; afilho@fmv.ulisboa.pt (A.L.A.F.); rjbbessa@fmv.ulisboa.pt (R.J.B.d.B.); 3 Laboratório Associado para Ciência Animal e Veterinária (AL4AnimalS), 1300-477 Lisboa, Portugal; 4 Centro de Pesquisa e Desenvolvimento de Zootecnia Diversificada, Instituto de Zootecnia, Rua Heitor Penteado, 56, Nova Odessa 13380-011, São Paulo, Brazil; rldcosta@sp.gov.br (R.L.D.d.C.); lecorreavet95@gmail.com (L.d.S.C.); 5 Instituto Federal Goiano (IF Goiano), Rua Sul Goiânia, 1371, Rio Verde 75906-750, Goiás, Brazil; josielzootecnista@gmail.com; 6 Center for Carbon Research in Tropical Agriculture (CCARBON), University of São Paulo, Alameda das Palmeiras, 11, Piracicaba 13416-823, São Paulo, Brazil

**Keywords:** CH_4_, microbial protein synthesis, residual feed intake, sustainability

## Abstract

Feed efficiency classification based on residual feed intake (RFI) and residual intake and gain (RIG) is widely used to identify biologically efficient animals, yet it remains unclear whether this classification reflects consistent differences in digestive, fermentative, and metabolic processes. This study evaluated the effects of RFI and RIG classification on nutrient intake, apparent digestibility, ruminal fermentation, nitrogen metabolism, microbial protein synthesis, and gaseous emissions in confined lambs. Thirty-eight weaned Texel ewe lambs underwent a 60-day performance test using an automated feed intake system and were classified as high-efficiency, neutral, or low-efficiency based on both indices. Animals were individually housed in respirometric chambers where emissions of methane, carbon dioxide, nitrous oxide, and ammonia were assessed by cavity ring-down spectroscopy; apparent digestibility was determined from total collections of feed, orts, faeces, and urine; microbial protein synthesis was estimated from urinary purine derivatives; and ruminal short-chain fatty acid profiles were determined by gas chromatography. Feed efficiency classification did not significantly affect body weight, nutrient intake, apparent digestibility, ruminal fermentation parameters, nitrogen balance, microbial protein synthesis, or greenhouse gas emissions. Principal component analysis revealed two major biological gradients related to nutrient intake and utilisation (42.9%) and ruminal fermentation and gaseous emissions (23.3%), together explaining 66.2% of total variance, but showing no clear separation among efficiency groups. These findings indicate that the digestive, fermentative, and nitrogen metabolism variables evaluated in this study did not account for the observed variation in feed efficiency. Because the regression underlying RIG explained little additional variation (R^2^ = 0.01), these conclusions primarily reflect feed efficiency as classified by RFI, suggesting that other physiological mechanisms may play a more important role in determining feed efficiency in confined Texel ewe lambs.

## Simple Summary

Selecting sheep for feed efficiency is an important strategy to reduce feed costs and the environmental footprint of livestock production. Two common efficiency indices, residual feed intake (a measure of how much an animal eats relative to its expected intake) and residual intake and gain (which also accounts for body weight gain), are widely used to rank animals from more to less efficient. However, in the present study, the regression model used to derive residual gain explained only a very small proportion of the variation in average daily gain (R^2^ = 0.01), meaning that the residual intake and gain classification largely reflected the inverse of residual feed intake. Even so, it remains unclear whether animals ranked differently by these indices actually differ in how they digest feed, ferment it in the rumen, use nitrogen, or emit greenhouse gases. To investigate this, 38 weaned Texel ewe lambs were ranked by these two efficiency indices over a 60-day performance test. Digestibility, rumen fermentation products, nitrogen metabolism, microbial protein synthesis, and greenhouse gas emissions were measured in individual chambers. No meaningful differences were found between efficiency groups for any of these traits. These results suggest that the biological processes evaluated here do not explain the variation captured by these indices, and that other mechanisms, yet to be identified, may drive differences in feed efficiency in this type of animal.

## 1. Introduction

The growing demand for sustainable livestock production systems, driven by global population growth and mounting environmental constraints, has intensified the need to improve resource-use efficiency while reducing greenhouse gas (GHG) emissions [1]. In this context, sheep production systems offer a useful model for evaluating strategies that simultaneously enhance productivity and mitigate environmental impacts, particularly those associated with emissions of enteric methane (CH_4_), nitrous oxide (N_2_O), and ammonia (NH_3_). Therefore, integrating animal performance, nutrient utilisation, and emission dynamics is essential for a comprehensive assessment of production efficiency and sustainability.

Feed efficiency remains a central trait in this framework because it directly influences feed costs, animal performance, and the environmental footprint per unit of product [2]. Metrics such as residual feed intake (RFI) and residual intake and gain (RIG) are widely used to classify animals by biological efficiency, enabling the identification of individuals who achieve similar or superior performance with lower feed intake [3,4]. However, the extent to which these classifications reflect consistent differences in digestive efficiency, metabolic processes, nutrient partitioning, and environmental impact remains unclear and may depend on diet composition, animal type, and experimental conditions [5]. Furthermore, reported relationships between feed efficiency, nitrogen utilisation, and GHG emissions have been inconsistent across studies.

Previous studies have suggested that feed-efficient animals may differ in nutrient utilisation, ruminal microbial activity, and fermentation characteristics [6]. Such differences could influence the production of short-chain fatty acids (SCFAs), microbial protein synthesis, and hydrogen-utilisation pathways in the rumen, thereby affecting enteric CH_4_ emissions. However, findings are not always consistent, and several studies have reported that differences in feed efficiency are not necessarily accompanied by detectable changes in fermentation profiles, nutrient digestibility, or CH_4_ production [7,8]. These inconsistencies highlight the need for integrated evaluations that simultaneously assess digestive, metabolic, and environmental responses associated with feed efficiency.

Nitrogen metabolism represents another important component of livestock sustainability because inefficient nitrogen utilisation increases environmental losses through urinary and faecal excretion. Quantifying the urinary excretion of purine derivatives enables the estimation of microbial protein synthesis and provides insights into how efficiently animals capture and utilise dietary nitrogen [9,10]. Consequently, evaluating nitrogen metabolism alongside ruminal fermentation and GHG emissions may improve understanding of the biological mechanisms associated with feed efficiency.

Recent advances in emission-monitoring technologies have facilitated the simultaneous evaluation of GHG emissions and animal performance under practical feeding conditions [11]. Integrating these measurements with data on intake, digestibility, ruminal fermentation, and nitrogen metabolism enables a more comprehensive assessment of the biological and environmental dimensions of feed efficiency.

We hypothesised that lambs divergent for RFI/RIG would differ in ruminal fermentation patterns, nitrogen partitioning, and GHG emissions. Therefore, the aim of this study was to evaluate the productive and environmental implications of feed efficiency in sheep by characterising nutrient intake, apparent nutrient digestibility, ruminal fermentation profiles, emissions of CH_4_, CO_2_, N_2_O, and NH_3_, as well as nitrogen balance and microbial protein synthesis. Additionally, multivariate analyses were used to integrate these variables and explore relationships among intake, digestion, metabolism, and GHG emissions in sheep divergent for feed efficiency.

## 2. Materials and Methods

### 2.1. Location and Facilities

The experiment was conducted at the sheep feedlot unit of the Instituto de Zootecnia (IZ), Agência Paulista de Tecnologia dos Agronegócios (APTA), affiliated with the Secretaria de Agricultura e Abastecimento do Estado de São Paulo, in Nova Odessa, São Paulo, Brazil (22°46′39″ S, 47°17′45″ W), and at the Laboratório de Nutrição Animal (LANA) of the Centro de Energia Nuclear na Agricultura (CENA), Universidade de São Paulo (USP), in Piracicaba, São Paulo, Brazil (22°23′31″ S, 47°38′57″ W). The experiment was conducted between November 2022 and February 2023. Throughout the feed efficiency trial, mean ambient temperature and relative humidity were 25.8 ± 1.68 °C and 69.3 ± 5.48%, respectively, while during the gas collection and digestibility trial, temperature and relative humidity inside the respirometric chambers averaged 26.3 ± 0.33 °C and 84.1 ± 2.51%, respectively.

### 2.2. Animals, Performance and Feed Efficiency

Thirty-eight Texel ewe lambs, with a mean initial age of approximately 138 days and a mean initial body weight of 32.2 ± 4.42 kg, were used in this study. The animals were sourced from a private flock at Fazendas União do Brasil, located in Buri, São Paulo, Brazil. Before the start of the experimental period, all animals underwent routine sanitary management, including vaccination and anthelmintic treatment, according to the farm health programme. Animals were clinically examined and confirmed to be healthy before entering the experiment. Upon arrival, the lambs were housed in a communal pen at the IZ feedlot facility, which was equipped with an automated feed-intake measurement system (Intergado^®^, Contagem, MG, Brazil) comprising nine feeding stations and two water troughs with integrated scales. The communal pen was located in a roofed, naturally ventilated facility with free access to fresh water throughout the experimental period. The system continuously recorded, on a 24-h basis, individual dry-matter intake (DMI), feeding and drinking visit frequency (with and without consumption), and body weight. Animals underwent a 14-day adaptation period, followed by a 60-day experimental period, during which they had ad libitum access to feed and water.

Daily intake and average daily gain (ADG) data were automatically compiled into electronic spreadsheets generated by the Intergado^®^ system. Based on these records, animals were classified into three groups according to residual feed intake (RFI) and residual intake and gain (RIG): high efficiency (RFI^−^ and RIG^+^), neutral (RFI± and RIG±), and low efficiency (RFI^+^ and RIG^−^). The symbols −, +, and ± indicate values below −0.5 SD, above +0.5 SD, and within ±0.5 SD of the population mean, respectively. The standard deviations (SDs) were 0.461 for RFI and 0.470 for RIG. This classification resulted in *n* = 10 high-efficiency, *n* = 18 neutral, and *n* = 10 low-efficiency animals (Appendix A).

Residual feed intake (RFI) was calculated according to the method proposed by Koch et al. [3] as the residual from the regression of dry matter intake (DMI) on average daily gain (ADG) and metabolic body weight (BW^0.75^) and expected DMI estimated from a multiple regression model:

DMI = β_0_ + β_1_ADG + β_2_BW^0.75^ + ε (1)

where DMI is dry matter intake, ADG is average daily gain, BW^0.75^ is metabolic body weight, β_0_ is the intercept, β_1_ and β_2_ are partial regression coefficients, and ε is the residual error term. Residual gain (RG) was calculated as the residual from the regression of ADG on DMI and BW^0.75^:

ADG = β_0_ + β_1_DMI + β_2_BW^0.75^ + ε (2)

Expected dry matter intake (DMIe) and expected average daily gain (ADGe) were obtained from the fitted regression equations. Residual feed intake (RFI) and RG were then calculated as the differences between observed and expected values:

RFI = DMI − DMIe(3)

RG = ADG − ADGe(4)

Residual intake and gain (RIG) was calculated according to Berry and Crowley [4]:

RIG = (−1 × RFI) + RG(5)

Positive RIG values indicate animals with lower feed intake and/or greater weight gain than expected, whereas negative values indicate poorer biological efficiency.

The descriptive characteristics of the high-efficiency, neutral, and low-efficiency groups, including body weight, dry matter intake, average daily gain, RFI, RG, and RIG, are summarized in Table 1.

### 2.3. Diet Composition

Throughout the experimental period, animals were fed a commercial total mixed ration (TMR, Table 2) formulated for confined sheep (RC Dieta Total Ovinos Confinamento^®^, Coopermota, Cândido Mota, SP, Brazil), at a forage-to-concentrate ratio of 15:85 (coast-cross hay:concentrate, DM basis).

### 2.4. Quantification of In Vivo Enteric CH_4_ Production

Following the feed efficiency test, enteric CH_4_ emissions were quantified using open-circuit respirometric chambers at LANA-CENA/USP, Piracicaba, SP, Brazil. The 38 animals were transported in groups of 10 from the IZ feedlot to the LANA facility in a vehicle adapted for animal transport. After each measurement period, the group was returned to the IZ, and the next group was allocated to the chambers for the subsequent collection period. The remaining 28 animals at the IZ continued under the same management conditions used during the performance test.

Each group of 10 animals was housed individually in respirometric chambers for three days, comprising one day of adaptation followed by two consecutive 24-h gas collection periods. Each collection period represented an integrated 24-h measurement, thereby capturing the complete diurnal cycle of gas production, including both daily feeding events. This two-day repeated-measurement design was adopted to ensure data repeatability, with day treated as a repeated within-animal factor in the statistical model. On the first day (adaptation), the polyethylene sealing panels on the lateral walls of the chambers were removed to facilitate air exchange with the external environment. On the second day, the panels were reinstalled, the measurement equipment was activated, and gas collection was initiated.

The respirometric chambers (157 × 71 × 167 cm; internal volume: 1.19 m^3^) consisted of adapted metabolic cages with metal frames and polyethylene sealing panels. Each chamber was equipped with a 5 cm-diameter inlet orifice on the front panel for ambient air entry and a corresponding 5 cm-diameter outlet orifice on the rear panel, connected via tubing to an exhaust pump that continuously removed gases from the chamber interior. Each chamber also contained individual feed and water troughs, a fan for internal air circulation, and a digital thermometer. Additional thermometers and fans were installed in the external environment surrounding the chambers to maintain adequate thermal and environmental conditions for animal welfare.

Prior to chamber allocation, animals were individually weighed (46.9 ± 4.91 kg) and identified. Body weight was recorded again at the end of the measurement period (45.0 ± 5.61 kg). Within the chambers, animals were offered the same commercial total mixed ration used during the feedlot period, provided twice daily: half the daily allowance in the morning (08:00–09:00 h) and the remaining half in the afternoon (15:00–16:00 h). Water was always available ad libitum. Feed was supplied by briefly opening the front panel of each chamber to minimise interference with gas collection. Orts were weighed daily, and feed allowance was adjusted according to each animal’s individual intake recorded during the feedlot period via the Intergado^®^ system.

Gas samples were collected using a peristaltic pump (Ismatec IPC 12, Ismatec, Wertheim, Germany) that continuously removed air from the chamber at a flow rate of 100 mL/min and delivered it into 5-L sampling bags lined with aluminium foil. To prevent sampling bags from exceeding their capacity, the bags were replaced at regular intervals throughout each 24-h collection period. After each collection cycle, 10 mL of gas from each bag was sampled in triplicate using a 20-mL syringe (Becton Dickinson Indústria Cirúrgica Ltd., Curitiba, Brazil) and stored in 10-mL evacuated tubes for subsequent analysis. Gas samples were analysed by gas chromatography (Shimadzu GC-2010, Tokyo, Japan) equipped with a flame ionisation detector and an HP-Molesieve capillary column (30 m × 0.53 mm × 25 µm). Methane concentrations were determined using a four-point calibration curve (0, 30, 90, and 120 mL/L) prepared from a certified CH_4_ standard (Praxair Industrial Gases, Osasco, Brazil; purity: 99.5%). Temperature (internal and external), relative humidity (internal and external), and airflow through the chamber (measured by an anemometer) were recorded at 3-h intervals throughout the collection period. The total gas volume extracted from each chamber was corrected to standard pressure and temperature (STP), and total enteric methane production was integrated over each complete 24-h collection period using the net CH_4_ concentration (%) and the total gas volume (L/day) removed from each chamber.

Methane emissions were expressed as total daily production (L/day) and as the mass of CH_4_ emitted per day (g/day). Additionally, emissions were normalised relative to animal body size and feed intake, including methane production per unit of body weight (g/kg BW), metabolic body weight (g/kg BW^0.75^), and dry matter intake (g/kg DMI).

### 2.5. Emissions of CH_4_, CO_2_, N_2_O and NH_3_

The daily production profiles of CH_4_, CO_2_, N_2_O, and NH_3_ were assessed using the same animals housed in the open-circuit respirometric chambers. Each chamber was connected via polytetrafluoroethylene (PTFE) tubing (4 mm internal diameter) to a 16-port gas distribution manifold (A0311, Picarro Inc., Santa Clara, CA, USA), which sequentially directed the gas from each chamber to a cavity ring-down spectroscopy (CRDS) analyser (G2508, Picarro Inc., Santa Clara, CA, USA). This system enabled simultaneous, real-time detection of CO_2_, CH_4_, and N_2_O concentrations (ppm; µmol/mol *v*/*v*) and NH_3_ concentrations (ppb; nmol/mol *v*/*v*) at an acquisition frequency of approximately 1 Hz. An additional sampling tube was used to continuously monitor background gas concentrations in the room housing the chambers.

Gas measurements were conducted sequentially across 10 animal chambers and one ambient reference chamber, with a sampling duration of three minutes per chamber, resulting in complete measurement cycles of 33 min. Continuous monitoring was carried out from 08:00 h to 06:00 h the following day, yielding approximately 22 h of data acquisition. This monitoring period encompassed both daily feeding events and was used to characterise the temporal emission profile of the gases rather than to estimate 24-h cumulative methane production, which was obtained independently from the integrated gas chromatography measurements. Internal temperature, relative humidity, and airflow (m/s) within each chamber were recorded five times throughout the day, and mean daily values were calculated for each parameter. The measurement protocol spanned three days per group of animals: one adaptation day followed by two consecutive 24-h collection periods. At the end of the first collection day, chambers were thoroughly cleaned, and faeces, urine, and feed residues were removed before measurements resumed on the second day.

Raw data from the gas analyser were processed in RStudio 9.5 [15] using the packages dplyr [16], tidyr [17], and lubridate [18]. To minimise the risk of cross-contamination between sequential chamber measurements, the first 60 s of each 3-min sampling session were excluded from analysis. The remaining two minutes of each session were used to calculate rolling mean gas concentrations, yielding one mean concentration per gas per chamber every 33 min. Over the 44-h monitoring period, a total of 76 measurement points per chamber were collected, yielding temporal gas concentration profiles for each animal.

Total gas emissions were estimated by calculating the area under the concentration–time curve (AUC) using numerical integration with the trapezoidal rule, approximating the integral as the sum of trapezoidal areas between consecutive measurements. Daily emissions were expressed as the mass of gas emitted per day (g/day) and normalised per unit of metabolic body weight (g/kg BW^0.75^/day) and per unit of dry matter intake (g/kg DMI), enabling comparisons among animals with different body sizes and intake levels.

### 2.6. Feed Intake and Apparent Digestibility

During the in vivo gas collection period, comprising one day of adaptation followed by two consecutive 24-h collection days, as described in Section 2.4, individual samples of offered feed, orts, faeces, and urine were collected daily from each animal on the two collection days; data from the adaptation day were excluded from intake and digestibility calculations. The diet, orts, and faeces were weighed on an electronic scale, and a representative 10% subsample of each material was stored at 4 °C. These samples were then thawed at room temperature, reweighed, and dried in a forced-ventilation oven at 55 °C to constant weight. They were then ground to pass a 1 mm sieve and subjected to chemical analysis to determine dry matter (DM; ID number 934.01), organic matter (OM; calculated by difference), mineral matter (MM; ID number 942.05), crude protein (CP; ID number 2001.11), and ether extract (EE; ID number 2003.5) [12]. Neutral detergent fibre (NDF; ID number 2002.04) and acid detergent fibre (ADF; ID number 973.18) were determined [14], and the coefficients of apparent digestibility were calculated [19], using the equation:

DX (%) = [(X intake − X faecal excretion)/X intake] × 100(6)

where DX represents the apparent digestibility of nutrient X.

### 2.7. Nitrogen Balance and Microbial Protein Synthesis

Urine was collected daily using graduated plastic containers that had been acidified with 100 mL of 10% H_2_SO_4_ to prevent ammonia volatilisation. Total individual urine volumes were recorded, and a 10% aliquot was collected to form a composite sample per animal, which was stored at –20 °C for subsequent determination of ammonia nitrogen, total nitrogen and purine derivatives. Nitrogen balance was determined for each animal based on the nitrogen content of the diet, orts, faeces and urine, analysed using the micro-Kjeldahl method [12]. Nitrogen retained was calculated as:

N retained (g/day) = N intake − N excretion(7)

where N intake corresponds to the nitrogen consumed and N excretion represents the sum of faecal and urinary nitrogen losses.

Microbial protein synthesis was estimated from urinary purine derivatives (PDs) following the protocol of Makkar and Chen [10], as described by Abdalla Filho et al. [20]. Prior to analysis, acidified urine samples were thawed at room temperature, homogenised by sonication for 5 min to resuspend particulate material, and aliquots were used for extraction and quantification of PD by high-performance liquid chromatography (HPLC) on an Agilent 1100 system equipped with a quaternary pump, degasser, autosampler, thermostat, photodiode array detector (UV-Vis), and a Zorbax ODS C18 column (250 × 4.6 mm; 5 µm particle size). Separation was performed using an ammonium phosphate buffer (0.0025 M) and methanol (95:5, *v*/*v*), with the column temperature maintained at 23 °C. Standard solutions of allantoin, creatinine, uric acid, xanthine, and hypoxanthine (500–1500 µM) were used to construct calibration curves, and oxypurinol served as the internal standard. Detection wavelengths were set at 225 nm (allantoin and creatinine), 267 nm (xanthine), 284 nm (uric acid), and 254 nm (hypoxanthine and oxypurinol). Microbial nitrogen (MN) absorbed in the small intestine was subsequently calculated from PD according to Makkar and Chen [10]:

MN = (PDA × 70)/(0.116 × 0.83 × 1000)(8)

where PDA represents absorbed purine derivatives (mmol/day/kg^0.75^).

Microbial protein synthesis efficiency (%) was calculated as the ratio between MN (g/day) and nitrogen intake (g/day) according to Chen and Gomes [21], representing the proportion of ingested nitrogen that was directed toward ruminal microbial protein synthesis:

MP Efficiency = (MN/N intake) × 100(9)

### 2.8. Short Chain Fatty Acids

At the end of the gas collection and digestibility period, ruminal fluid samples were collected at CENA/USP before the morning feeding via a silicone oesophageal tube connected to a syringe. Tube insertion was performed by trained personnel to minimise oesophageal mucosal injury, and animals were monitored for signs of discomfort throughout the procedure. The first collected fluid was discarded to minimise salivary contamination. Approximately 60 mL of ruminal fluid was then obtained from each animal, transferred to glass tubes, and stored at −20 °C until determination of short-chain fatty acids (SCFAs). SCFA concentrations were determined by gas chromatography, following Palmquist and Conrad [22] with adaptations by Lima et al. [23]. Results were expressed as total concentration (mmol/L) and as molar proportions (%) of individual acids, including acetate (C2; acetic acid), propionate (C3; propionic acid), butyrate (C4; butyric acid), isovalerate (IC5; isovaleric acid), and valerate (C5; valeric acid). The acetate-to-propionate ratio (C2:C3) was also calculated as an indicator of ruminal fermentation balance.

### 2.9. Statistical Analysis

Statistical analyses were performed using SAS^®^ 9.4 software [24] with the PROC MIXED procedure. Data on productive performance, rumen fermentation parameters, and nitrogen metabolism variables were merged into a single dataset using individual animal identification. Animals were classified into three categories based on residual feed intake (RFI) and residual intake and gain (RIG): high efficiency, low efficiency, and neutral.

For variables obtained during the respiration-chamber phase, including nutrient intake, apparent digestibility, nitrogen metabolism, microbial protein synthesis, ruminal fermentation, and gas-emission variables, measurements collected over the two consecutive 24-h collection days were averaged for each animal before statistical analysis. Consequently, each animal contributed a single observation for each response variable, and no repeated-measures structure was applied. These variables were analysed using linear mixed models that included feed-efficiency class and chamber measurement period as fixed effects, with period serving as a blocking factor to account for potential temporal variation among the four sequential measurement batches. Variables obtained outside the respiration-chamber phase were analysed using models that included feed-efficiency class as the fixed effect. The individual animal was considered the experimental unit in all analyses. Models were fitted using restricted maximum likelihood (REML). Least-squares means were estimated for each feed-efficiency class and compared using pairwise differences (PDIFF). In addition, a single-degree-of-freedom planned contrast was defined a priori to compare the high-efficiency and low-efficiency groups, based on the hypothesis that the greatest divergence in feed-efficiency classification would be most likely to reveal biologically relevant differences. Statistical significance was declared at *p* < 0.05. In addition to *p*-values, results for the pre-specified planned contrast between high- and low-efficiency animals are presented as the estimated difference (high efficiency minus low efficiency), together with its 95% confidence interval (95% CI) and Hedges’ g as a standardised effect size. To compare methane measurements obtained by gas chromatography and cavity ring-down spectroscopy, the Pearson correlation coefficient was calculated using individual-animal data. Principal component analysis (PCA) was performed to explore patterns of association among variables related to ruminal fermentation, nutrient intake, digestibility, and metabolic responses using the vijPlots module [25] implemented in jamovi software [26]. Before the PCA, a Pearson correlation matrix was examined to assess multicollinearity among variables. The Pearson correlation coefficient between RFI and RIG was also calculated to quantify the relationship between the two feed-efficiency indices. Variables showing high collinearity (r > 0.90) were considered redundant and excluded. Variables representing alternative expressions of the same biological parameter, such as values adjusted for metabolic body weight or intake, were also removed to avoid duplication of information. The final dataset included representative variables from each biological domain, including fermentation, intake, digestibility, and metabolism. All variables were standardised to a mean of 0 and a standard deviation of 1, and the PCA was conducted using the correlation matrix. The number of components retained for interpretation was determined based on eigenvalues greater than 1 and the cumulative proportion of explained variance. The first two principal components (PC1 and PC2) were selected for graphical representation and interpretation. Loadings with absolute values ≥ 0.40 were considered relevant for interpreting the contribution of each variable, following the criterion recommended by Jolliffe [27]. Results were visualised using a component plot (loadings plot) and an observation plot (scores plot).

## 3. Results

### 3.1. Animals, Nutrient Intake, Apparent Digestibility, Nitrogen Metabolism and Microbial Protein Synthesis

**Figure 1:**
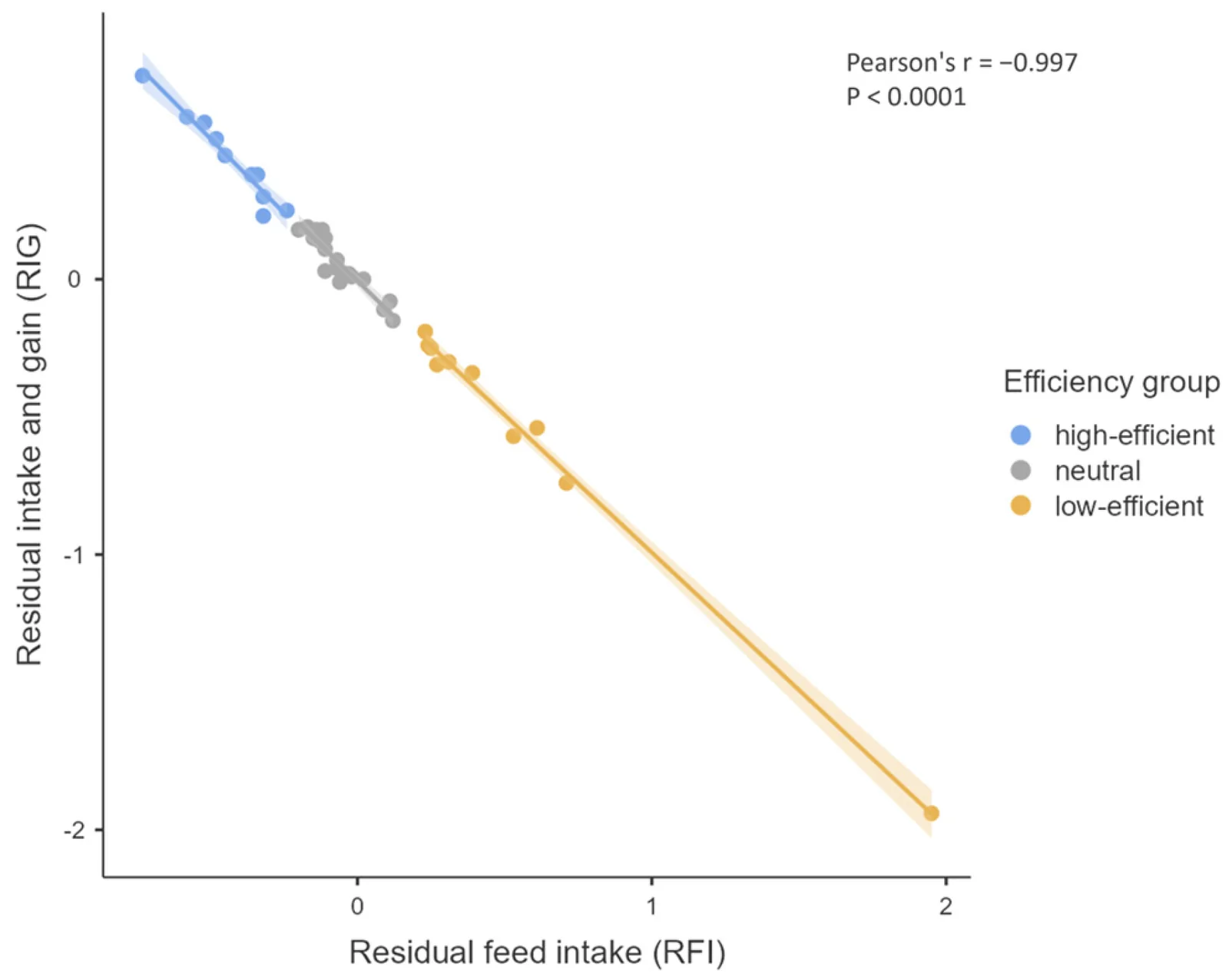
Relationship between residual feed intake (RFI) and residual intake and gain (RIG) in the 38 Texel ewe lambs. Each point represents one animal and is coloured according to feed efficiency classification (high efficiency, neutral, and low efficiency). Solid lines represent the fitted linear regressions within each group, and shaded areas indicate the corresponding 95% confidence intervals. The two feed efficiency indices were almost perfectly inversely correlated (Pearson’s r = −0.997, *p* < 0.0001).

The regression model used to calculate residual feed intake explained 56% of the variation in dry matter intake (R^2^ = 0.56), whereas the regression underlying residual gain explained only 1% of the variation in average daily gain (R^2^ = 0.01). Consistent with this limited contribution of residual gain, RFI and RIG were almost perfectly inversely correlated in the study population (Pearson’s r = −0.997, *p* < 0.0001; Figure 1), indicating that, under the conditions of the present study, RIG provided little additional variation beyond that already captured by RFI.

Body weight, nutrient intake, and apparent digestibility were not affected by feed efficiency classification (*p* > 0.05; Table 3). Similarly, retained N, microbial protein (MP) synthesis, and MP production efficiency were not affected (*p* > 0.05; Table 3). Although chamber measurement period affected NDF intake (expressed as g/d and relative to body weight) (*p* ≤ 0.042) and MP synthesis (*p* = 0.013), adjustment for this source of variation did not alter the absence of differences among feed-efficiency classes, and the planned contrast between high- and low-efficiency animals remained non-significant for all variables.

### 3.2. Short-Chain Fatty Acid

Short-chain fatty acid (SCFA) production was also not influenced by feed efficiency classification (*p* > 0.05; Table 4). Likewise, chamber measurement period did not affect total SCFA concentration, individual SCFA concentrations, molar proportions, or the acetate-to-propionate ratio (*p* > 0.05). Total SCFA concentration and molar proportions of individual fatty acids were similar across groups (Table 4).

### 3.3. Greenhouse Gas Emissions

Methane emissions measured by both the Picarro system and gas chromatography were not affected by feed efficiency classification (*p* > 0.05; Table 5). Likewise, chamber measurement period did not affect any gas emission variable (*p* > 0.05). Absolute CH_4_ production, as well as CH_4_ expressed relative to dry matter intake (DMI) and to metabolic body weight (BW^0.75^), showed similar values across groups. Likewise, feed efficiency classification did not affect the CH_4_:CO_2_ ratio, N_2_O emissions, or NH_3_ emissions (*p* > 0.05; Table 5). Emission variables were generally comparable across groups, regardless of the unit of expression.

### 3.4. Principal Component Analysis

**Figure 2:**
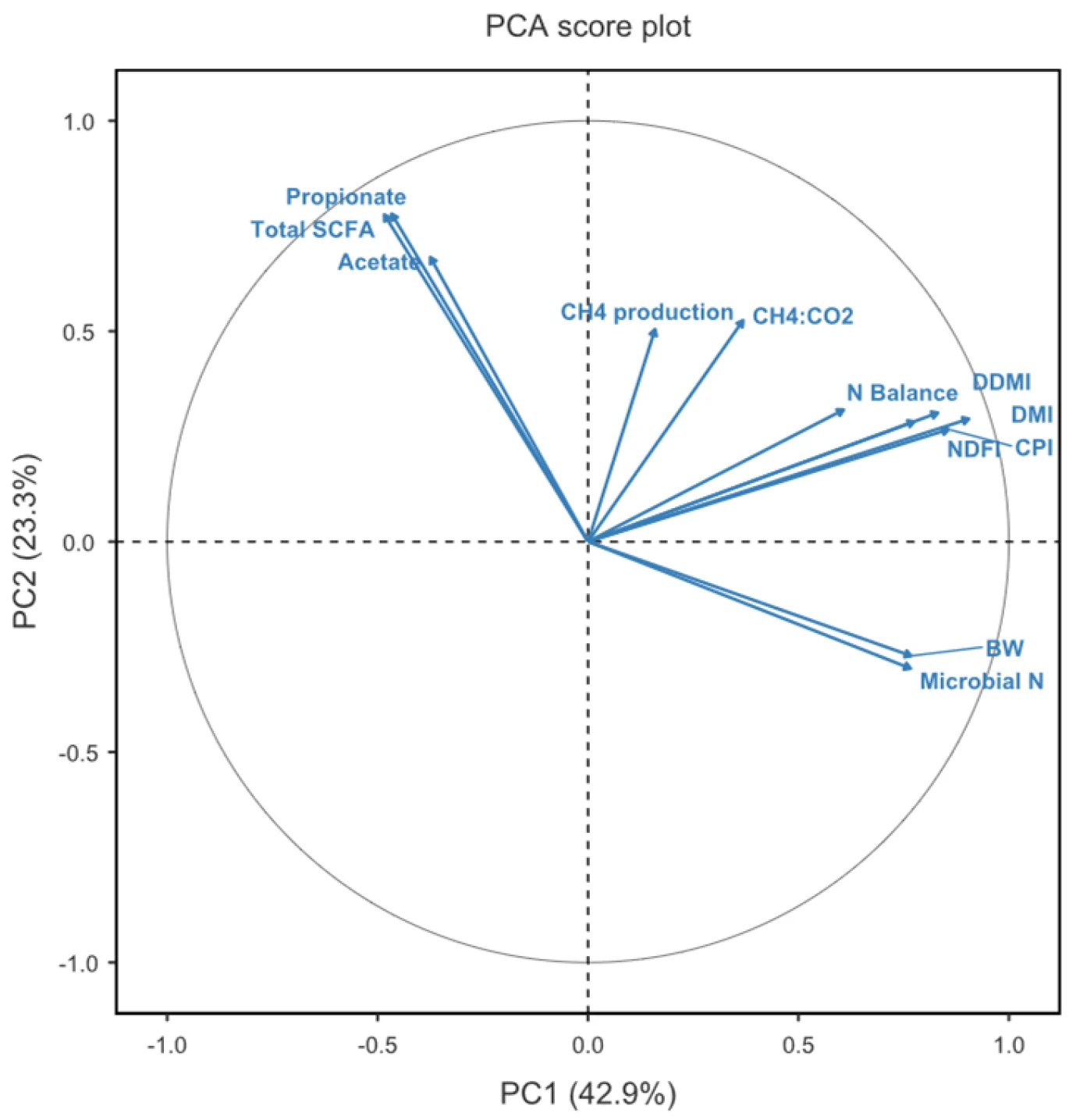
Principal Component Analysis (PCA) loading plot showing the contribution of the evaluated variables to the first two principal components (PC1 = 42.9% and PC2 = 23.3% of the total variance explained). Vectors represent the direction and magnitude of each variable’s contribution to the principal components. Variables pointing in the same direction are positively associated, whereas variables pointing in opposite directions are negatively associated. The distance from the origin reflects the strength of the contribution of each variable to the multivariate structure represented by the first two principal components.

Principal component analysis retained three components with eigenvalues greater than 1.0, together explaining 78.5% of the total variance. Because the first two principal components explained 66.2% of the total variance, they were used for graphical representation and interpretation (Figure 1 and Figure 2). The first principal component (PC1) explained 42.9% of the total variance and was primarily associated with nutrient intake and utilisation variables, including dry matter intake (DMI), digestible dry matter intake (DDMI), neutral detergent fibre intake (NDFI), crude protein intake (CPI), microbial nitrogen production (Microbial N), nitrogen balance (N balance), and body weight (BW). The second principal component (PC2) explained 23.3% of the total variance and was primarily associated with ruminal fermentation characteristics and gaseous emissions, including total short-chain fatty acids (Total SCFA), acetate, propionate, methane production (CH4 production), and the methane:carbon dioxide ratio (CH_4_:CO_2_).

**Figure 3:**
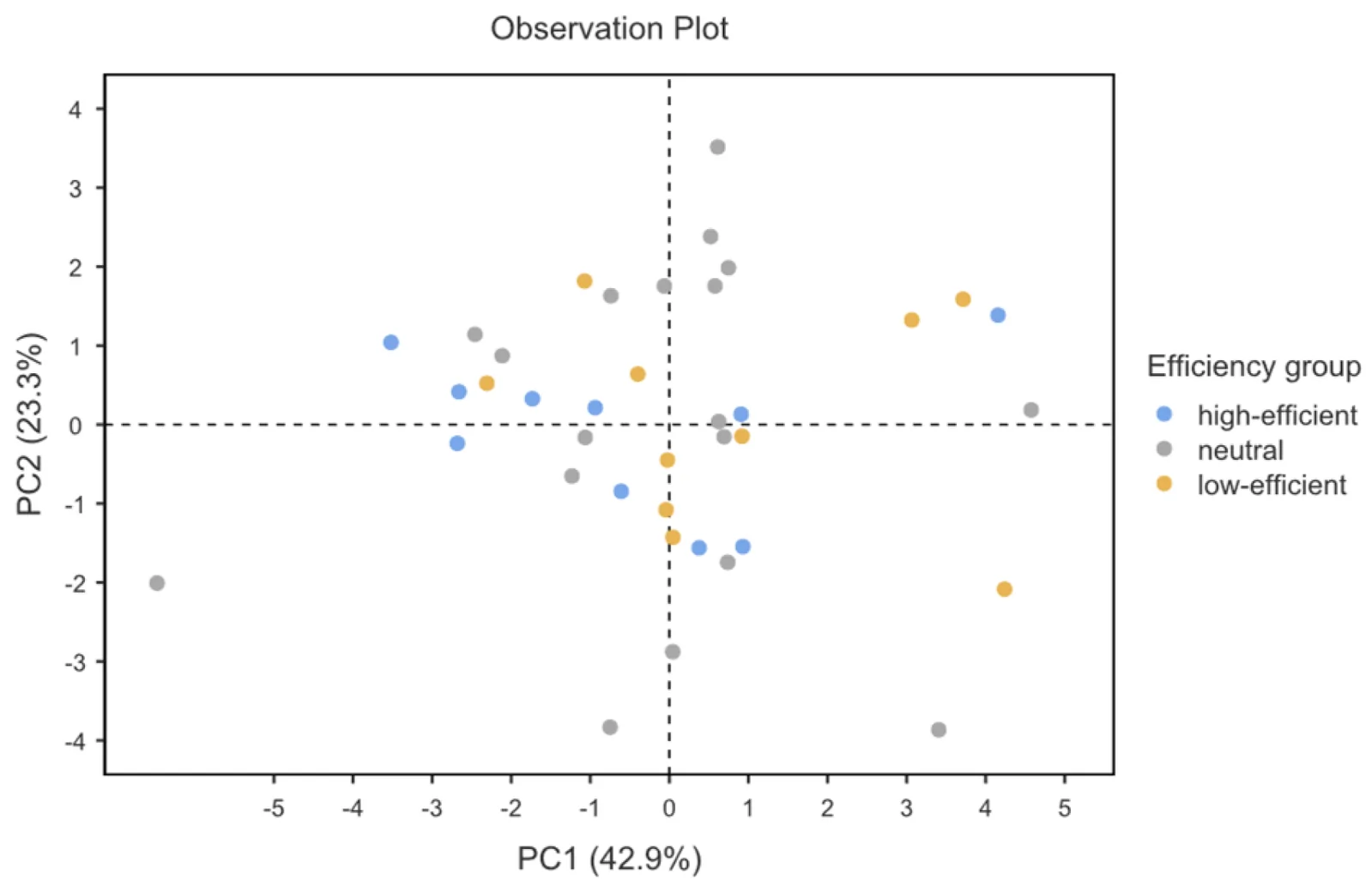
Principal Component Analysis (PCA) score plot of individual observations based on the first two principal components (PC1 = 42.9% and PC2 = 23.3%). Together, the first two principal components explained 66.2% of the total variance. Each point represents an individual animal. The distribution of observations reflects similarities in the multivariate profiles of nutrient intake, digestibility, nitrogen metabolism, and ruminal fermentation variables, with points located closer together indicating greater similarity among animals.

The loading plot (Figure 2) showed a clear separation between variables related to nutrient intake and utilisation, which were positively associated with PC1, and variables associated with ruminal fermentation and gaseous outputs, which were primarily associated with PC2. The score plot (Figure 3) illustrates the multivariate distribution of individual animals across these two major sources of variation. In the score plot (Figure 3), individual animals were distributed continuously across both principal components, with substantial overlap among the feed efficiency classifications (high efficiency, low efficiency, and neutral). No clear separation among groups was observed, indicating that the evaluated variables did not clearly discriminate animals according to feed-efficiency classification.

## 4. Discussion

The present study aimed to assess whether feed efficiency classification was associated with differences in nutrient utilisation, ruminal fermentation, N metabolism, and GHG emissions in Texel ewe lambs. We hypothesised that lambs divergent for RFI/RIG would differ in these parameters. However, the results largely failed to support this hypothesis. Animals classified as high-efficiency, low-efficiency, or neutral exhibited remarkably similar physiological and metabolic responses across most of the evaluated variables. Moreover, the effect sizes were generally small to moderate, and the 95% confidence intervals largely overlapped zero, supporting the interpretation that the evaluated traits did not show consistent or biologically meaningful differences between high- and low-efficiency animals. Collectively, these findings indicate that differences in feed efficiency, as defined in the present study, were not accompanied by detectable changes in the measured ruminal digestive and metabolic variables. This lack of association reflects the specific variables assessed in this study rather than excluding a broader role for digestive or ruminal physiology in determining feed efficiency, which may involve mechanisms not captured by the traits evaluated here.

Although the RFI model showed moderate explanatory power (R^2^ = 0.56), the regression model underlying RG explained only a small proportion of the variation in average daily gain (R^2^ = 0.01). R^2^ values reported for RFI and RG originate from two distinct regression models—DMI regressed on ADG and BW^0.75^ (underlying RFI), and ADG regressed on DMI and BW^0.75^ (underlying RG)—and therefore should not be interpreted as directly comparable measures of the predictive performance of these feed efficiency indices. By definition, RFI and RG are calculated as residuals of their respective regression models and are phenotypically independent of the predictor variables. Consequently, the low R^2^ observed for the ADG model reflects the weak phenotypic relationship between growth rate and feed intake/body weight in this population rather than a limitation of the efficiency classification. Furthermore, RIG was not used as an alternative to RFI, but as a composite index derived from both RFI and RG, allowing animals to be classified using complementary measures of feed efficiency [3,4,28]. However, because the regression underlying RG explained only a very small proportion of the variation in ADG, the contribution of residual gain to the composite index was minimal. This interpretation is supported by the almost perfect inverse correlation observed between RFI and RIG in the present dataset (Pearson’s r = −0.997, *p* < 0.0001), indicating that RIG was driven predominantly by the RFI component under the conditions of this study.

No differences were observed in BW, nutrient intake, or apparent digestibility among feed efficiency groups. This finding is particularly relevant, as it demonstrates that all animals were exposed to a comparable nutrient supply and exhibited similar digestive capacity. Therefore, differences in feed efficiency are unlikely to be explained by variations in feed intake or nutrient digestibility. This is consistent with previous studies reporting that RFI and related efficiency metrics are often independent of intake level and digestibility, suggesting that other physiological mechanisms contribute to variation in feed efficiency [3,5,7,28]. Similarly, it has been reported that sheep divergent for feed efficiency may exhibit comparable intake while differing in overall efficiency [8].

Consistent with the lack of differences in intake and digestibility, ruminal fermentation patterns were largely unaffected by feed efficiency classification. Total SCFA concentration and the molar proportions of individual fermentation end-products were similar among groups. This stability in the fermentation end-products measured suggests that the ruminal fermentation variables assessed here did not differ appreciably across feed efficiency classifications, although this does not exclude the possibility that other, unmeasured aspects of ruminal metabolism contributed to the observed efficiency differences. Given the central role of fermentation pathways in determining substrate availability for methanogenesis [29,30,31], these findings provide an important mechanistic basis for the observed CH_4_ results. Moreover, a recent meta-analysis demonstrated that the effects of dietary rumen-modulating strategies on fermentation characteristics and CH_4_ production are not necessarily parallel, highlighting the complexity of ruminal metabolic regulation [32].

Nitrogen metabolism also remained unaffected by feed efficiency classification. Nitrogen balance, microbial protein production, and microbial protein synthesis efficiency were similar across groups, indicating comparable nitrogen utilisation and microbial growth efficiency in the rumen. This suggests that the nitrogen-related variables evaluated here did not capture differences in ruminal microbial activity between feed efficiency groups, rather than indicating that microbial activity itself was unrelated to feed efficiency. These findings are consistent with the established role of purine derivatives as reliable indicators of microbial protein synthesis and nitrogen utilisation efficiency [5,9,10].

Methane emissions, measured using both the Picarro system and gas chromatography, were not affected by feed efficiency classification. Absolute CH_4_ production, as well as CH_4_ emissions expressed relative to DMI and BW^0.75^, were consistent across all groups. Although gas chromatography and cavity ring-down spectroscopy differed in their absolute methane estimates, the measurements obtained by the two methods were moderately correlated across individual animals (Pearson’s r = 0.515, *p* = 0.0009). It should be noted, however, that Pearson correlation evaluates association rather than analytical agreement between measurement methods. Because the objective of the present study was to compare feed-efficiency groups rather than to compare the analytical performance of the two methods, a formal agreement analysis was beyond the scope of this study. Nevertheless, both methods consistently supported the same biological conclusion, indicating the absence of an effect of feed-efficiency classification on methane emissions. The absence of differences in CH_4_ emissions is consistent with the similarity observed in nutrient intake, digestibility, and ruminal fermentation patterns. Since enteric CH_4_ production is primarily driven by substrate availability and fermentation pathways [33,34,35], the lack of variation in these factors explains the absence of differences in CH_4_ output among feed efficiency groups.

It is noteworthy that the CH_4_ yields observed in the present study were considerably lower than those generally reported for sheep, including values from respiration chamber studies under high-concentrate feeding conditions (~18–24 g CH_4_/kg DMI) [36] and those summarised in a recent meta-analysis of feedlot sheep, in which control treatments typically ranged from approximately 20 to 29 g CH_4_/kg DMI [37]. The highly fermentable nature of the experimental diet likely contributed to these relatively low values. Animals received a diet containing approximately 81% concentrate ingredients, relatively low NDF (36.83%), and monensin sodium, all of which are recognised to reduce methane production by favouring propionate formation and decreasing hydrogen availability for methanogenesis [38]. Nevertheless, these dietary characteristics alone are unlikely to fully explain the exceptionally low CH_4_ yields observed in the present study, as previous respiration chamber studies with sheep receiving similarly high-concentrate diets have generally reported substantially greater methane yields. Because methane production in the present experiment was quantified by integrating emissions over complete 24-h respiration chamber collection periods encompassing the full diurnal cycle and both daily feeding events, the low methane yields observed here cannot readily be attributed to incomplete daily sampling. The reasons for these comparatively low methane yields therefore remain uncertain and may reflect methodological differences among studies or other experimental factors that warrant further investigation.

Previous studies have reported inconsistent relationships between feed efficiency and enteric CH_4_ production. While some studies have observed lower CH_4_ emissions in more efficient animals due to improved energy utilisation and reduced substrate availability for fermentation [39,40,41], others have demonstrated weak or inconsistent associations between feed efficiency and CH_4_ emissions [42]. The present results support the latter observations, suggesting that enteric CH_4_ emissions alone may not be a reliable predictor of feed efficiency.

Similarly, NH_3_ and N_2_O emissions were not affected by feed efficiency classification. The lack of effect on gaseous nitrogen losses is consistent with the observed stability of nitrogen metabolism across groups and with previous descriptions of nitrogen dynamics in ruminant production systems [43].

The PCA results indicate that the dataset variability was structured along two main biological gradients: one associated with nutrient intake and utilisation (PC1), and another related to ruminal fermentation and metabolic activity (PC2), as commonly described in multivariate analyses of complex biological systems [27]. Despite this structured relationship among variables, no clear clustering of animals according to feed efficiency was observed. This overlap indicates that the evaluated variables were insufficient to distinguish animals by efficiency group and reinforces the multifactorial nature of feed efficiency, as similar patterns have been reported in multivariate studies, in which efficiency phenotypes exhibit continuous rather than discrete biological variation [44].

Collectively, the findings of overlap among efficiency groups suggest that the biological mechanisms underlying variation in feed efficiency were not captured by the ruminal and metabolic variables evaluated in the present study. From a practical perspective, the absence of an association between feed efficiency and CH_4_ production suggests that improving feed efficiency alone may not necessarily reduce enteric CH_4_ emissions under similar dietary and management conditions. This observation aligns with studies reporting weak or inconsistent genetic correlations between feed efficiency and CH_4_ production [45,46,47] and underscores the continued importance of nutritional strategies that specifically target ruminal methanogenesis [48]. The absence of a molecular fermentative signature in high-efficiency animals is consistent with the proteomic profiles reported for this same population, in which differences in plasma protein abundance were primarily associated with redox balance, metal ion homeostasis, and proteolytic regulation rather than with pathways directly linked to methanogenesis or substrate fermentation [49].

Some limitations should be acknowledged. The relatively small number of animals in the extreme feed-efficiency groups (*n* = 10) may have limited the statistical power to detect subtle biological differences, particularly for butyrate, N_2_O, and NH_3_, which showed the lowest *p*-values among the evaluated variables despite not reaching statistical significance. These observations should therefore be regarded as exploratory and interpreted with caution until confirmed in studies with larger sample sizes. Furthermore, the use of only Texel ewe lambs receiving a single commercial total mixed ration limits extrapolation of the present findings to other breeds, sexes, production systems, and dietary conditions and precludes assessment of diet × feed-efficiency interactions. Feed efficiency classification was established during the ad libitum feeding period, whereas ruminal fermentation, nitrogen metabolism, nutrient digestibility, and gas exchange measurements were subsequently performed under the standardised feeding conditions required by the respirometric chambers. Although this approach ensured comparable experimental conditions and minimised variation in feed intake during physiological measurements, it may also have reduced the expression of behavioural and metabolic differences associated with voluntary feed intake, thereby attenuating physiological differences between feed-efficiency groups. Consistent with this possibility, dry matter intake declined by approximately 15–20% relative to feedlot baseline levels after animals were transferred to the respirometric chambers (Table 1 and Table 3), reflecting a transient reduction in feed intake associated with confinement and the adaptation period. Although the present study was not specifically designed to formally test whether the magnitude of this reduction differed among feed-efficiency groups, no obvious differences among groups were apparent from the observed intake values. In addition, although the average chamber temperature (26.3 °C) was similar to that recorded during the feed-efficiency trial, the higher relative humidity inside the respirometric chambers (84.1%) may have increased the animals’ thermal load and contributed to the reduction in voluntary dry matter intake observed during chamber measurements. Consequently, measurements obtained under chamber conditions should be interpreted as reflecting responses under standardized experimental conditions rather than unrestricted feedlot conditions. Future studies should consider a longer adaptation period before gas collection to allow feeding behaviour to stabilise more completely. In addition, all animals received the same highly digestible, high-concentrate commercial diet containing monensin sodium. Although this ensured uniform nutritional management across treatments and did not compromise comparisons among feed-efficiency groups, both the dietary characteristics and the inclusion of monensin may have reduced inter-animal variation in ruminal fermentation, methane production, and associated metabolic responses, thereby limiting the physiological divergence among feed-efficiency classifications. Future studies evaluating animals under more challenging nutritional conditions, including higher-forage diets and the absence of ionophores, may help determine whether greater physiological differentiation emerges among feed-efficiency phenotypes.

Finally, the ruminal microbiota was not characterised, and microbial shifts or functional differences associated with feed efficiency may therefore have remained undetected. Beyond fermentation end-products, other physiological mechanisms not evaluated here have also been implicated in feed-efficiency variation. Mitochondrial function and efficiency of oxidative phosphorylation have been associated with RFI in cattle, with more efficient animals often exhibiting greater hepatic mitochondrial ATP synthesis and lower oxidative stress [28,50,51]. Likewise, metabolomic studies have identified circulating biomarkers, including branched-chain amino acids, acylcarnitines, and lipid-related metabolites, that differentiate efficiency phenotypes independently of digestive and fermentative traits [52,53]. Systemic physiological processes, such as protein turnover, immune activation, and thermoregulatory costs, have also been proposed as contributors to variation in feed efficiency [28]. Together with the plasma proteomic differences previously reported for this same population, which were primarily related to redox balance and metal ion homeostasis rather than digestive or fermentative pathways [49], these findings reinforce that feed efficiency is a multifactorial trait. Future studies integrating metagenomic, metabolomic, proteomic, and whole-animal physiological approaches may provide a more comprehensive understanding of the biological mechanisms underlying feed efficiency in ruminants.

## 5. Conclusions

The present study found no evidence that lambs divergent for RFI/RIG differ markedly in ruminal fermentation, nitrogen partitioning, or greenhouse gas emissions. Feed efficiency classification was not accompanied by consistent changes in digestive, fermentative, nitrogen-related, or emission variables, indicating that the variables measured did not explain the observed variation in feed efficiency. This lack of association suggests that, under the dietary and management conditions evaluated, feed efficiency is regulated by mechanisms not captured by the traits assessed here, reinforcing the view that it results from multiple, potentially interacting physiological pathways rather than from any single ruminal, digestive, or nitrogen-metabolism trait. In addition, the absence of a clear relationship between feed efficiency and CH_4_ emissions indicates that improvements in feed efficiency cannot be assumed to reduce enteric CH_4_ emissions under similar production conditions. Strategies aimed at mitigating greenhouse gas emissions from lamb production should therefore not rely solely on feed-efficiency classification but may require complementary nutritional, microbial, or rumen-targeted approaches.

## Figures and Tables

**Table 1:** Characteristics of the feed efficiency groups according to body weight, dry matter intake, average daily gain, residual feed intake (RFI), residual gain (RG), and residual intake and gain (RIG).

Parameter	High Efficiency (*n* = 10)	Neutral (*n* = 18)	Low Efficiency (*n* = 10)
BW^0.75^ (kg)	15.75 ± 0.77 (14.64 to 17.30)	15.78 ± 1.36 (13.10 to 18.18)	15.96 ± 1.70 (13.39 to 19.18)
DMI (kg/d)	1.047 ± 0.136 (0.880 to 1.256)	1.144 ± 0.175 (0.874 to 1.428)	1.195 ± 0.173 (0.831 to 1.424)
ADG (kg/d)	0.261 ± 0.027 (0.220 to 0.301)	0.240 ± 0.039 (0.145 to 0.301)	0.239 ± 0.038 (0.175 to 0.297)
RFI	−0.434 ± 0.148 (−0.734 to −0.243)	−0.064 ± 0.095 (−0.198 to 0.124)	0.549 ± 0.521 (0.233 to 1.954)
RG	0.007 ± 0.037 (−0.081 to 0.051)	−0.004 ± 0.036 (−0.089 to 0.059)	0.006 ± 0.037 (−0.042 to 0.063)
RIG	0.440 ± 0.164 (0.235 to 0.743)	0.061 ± 0.106 (−0.148 to 0.192)	−0.543 ± 0.521 (−1.939 to −0.189)

Values are presented as mean ± standard deviation (minimum–maximum). Animals were classified as high-efficiency, neutral, or low-efficiency according to RFI and RIG values using ±0.5 standard deviations from the population mean. BW^0.75^ = metabolic body weight; DMI = dry matter intake; ADG = average daily gain; RFI = residual feed intake; RG = residual gain; RIG = residual intake and gain; d = day.

**Table 2:** Chemical composition and list of ingredients of the total mixed ration provided to animals throughout the experimental period.

Parameter	Content
Dry matter (%)	90.76
Ash (%)	6.77
Crude protein (%)	18.91
Neutral detergent fiber (%)	36.83
Acid detergent fiber (%)	18.67
Ether extract (%)	4.04
Lignin (%)	5.57
Ingredients: Corn (45.29%), Cottonseed meal (16%), Sorghum (12%), Extruded whole soybeans (4%), Wheat bran (4%), Coast-cross grass hay (*Cynodon dactylon*) (15%), Calcitric limestone (2%), Dicalcium phosphate (0.14%), Sodium bicarbonate (0.75%), Ammonium chloride (0.50%), Vitamin–mineral premix (0.10%), Monensin sodium (0.01%), Preservative additives (0.10%).	

Values expressed on a dry matter basis at 105 °C. Chemical analysis was performed at the Laboratório de Nutrição Animal (LANA), Centro de Energia Nuclear na Agricultura (CENA/USP), following the analytical procedures described by [12,13,14].

**Table 3:** Effect of feed efficiency classification on body weight, nutrient intake and digestibility, retained N, microbial protein production and efficiency.

Parameter	High Efficiency	Neutral	Low Efficiency	*p*-Value	Difference (High − Low, 95% CI)	Hedges’ g
BW (kg)	44.7 ± 1.57	46.1 ± 1.17	47.0 ± 1.57	0.588	−2.28 (−6.80 to 2.24)	−0.45
BW^0.75^ (kg)	17.2 ± 0.45	17.6 ± 0.33	17.9 ± 0.45	0.597	−0.64 (−1.95 to 0.65)	−0.44
Intake (g/d)						
DM	886 ± 48.53	916 ± 36.17	973 ± 48.53	0.444	−86.6 (−225.9 to 52.6)	−0.57
OM	827 ± 45.74	854 ± 34.09	906 ± 45.74	0.469	−78.4 (−209.8 to 52.8)	−0.55
CP	165 ± 9.77	171 ± 7.28	187 ± 9.77	0.272	−21.7 (−49.7 to 6.36)	−0.72
NDF	310 ± 22.95	343 ± 17.08	361 ± 22.68	0.441	−50.8 (−116.2 to 14.4)	−0.61
DDM	656 ± 40.48	654 ± 30.17	706 ± 40.48	0.560	−49.5 (−165.7 to 66.7)	−0.39
DOM	626 ± 38.73	625 ± 28.87	673 ± 38.73	0.583	−46.1 (−175.3 to 64.1)	−0.39
DCP	131 ± 7.84	135 ± 5.84	148 ± 7.84	0.298	−16.3 (−38.8 to 6.17)	−0.66
Intake (g/d/kg BW)						
DM	19.8 ± 0.95	19.9 ± 0.71	20.8 ± 0.95	0.700	−1.01 (−3.75 to 1.73)	−0.36
OM	18.4 ± 0.88	18.5 ± 0.66	19.3 ± 0.88	0.716	−0.88 (−3.43 to 1.66)	−0.34
CP	3.70 ± 0.20	3.73 ± 0.15	4.01 ± 0.20	0.473	−0.30 (−0.88 to 0.27)	−0.54
NDF	6.91 ± 0.48	7.45 ± 0.36	7.80 ± 0.48	0.428	−0.88 (−2.27 to 0.49)	
DDM	14.6 ± 0.78	14.1 ± 0.58	15.0 ± 0.78	0.646	−0.41 (−2.67 to 1.84)	−0.18
DOM	13.9 ± 0.75	13.5 ± 0.56	14.3 ± 0.75	0.675	−0.37 (−2.53 to 1.79)	−0.16
DCP	2.94 ± 0.156	2.93 ± 0.116	3.17 ± 0.156	0.440	−0.23 (−0.68 to 0.21)	−0.48
DNDF	7.16 ± 0.518	7.34 ± 0.386	7.87 ± 0.518	0.600	−0.70 (−2.19 to 0.78)	−0.41
Digestibility (g/kg)						
DM	741 ± 22.1	710 ± 16.5	725 ± 22.1	0.529	16.1 (−47.4 to 79.7)	0.24
OM	757 ± 21.6	727 ± 16.1	742 ± 21.6	0.551	15.1 (−47.0 to 77.3)	0.23
CP	795 ± 15.6	786 ± 11.6	788 ± 15.6	0.897	7.0 (−37.8 to 52.0)	0.14
NDF	468 ± 53.5	359 ± 39.9	408 ± 53.5	0.274	59.7 (−93.9 to 213.4)	0.40
N balance and MP						
N retained (g)	16.70 ± 1.470	16.01 ± 1.095	19.28 ± 1.470	0.212	−2.57 (−6.79 to 1.64)	−0.56
MP Synthesis	3.52 ± 0.039	3.56 ± 0.029	3.56 ± 0.039	0.650	−0.04 (−0.15 to 0.06)	−0.43
MP Efficiency	22.31 ± 2.290	24.78 ± 1.707	19.18 ± 2.290	0.159	3.13 (−3.44 to 9.71)	0.62

*n* = 10 (high efficiency), 18 (neutral), 10 (low efficiency). Values expressed as LSMeans ± SEM adjusted for chamber measurement period. BW—body weight, BW^0.75^—metabolic body weight, DM—dry matter, OM—organic matter, CP—crude protein, NDF—neutral detergent fiber, DDM—digestible DM, DOM—digestible organic matter, DCP—digestible CP, DNDF—digestible NDF. MP—microbial protein. d—day. *p*-values refer to the overall effect of feed efficiency classification. The estimated difference between high- and low-efficiency animals (high − low) is presented with its 95% confidence interval (95% CI), and Hedges’ g is reported as the standardised effect size for this planned comparison.

**Table 4:** Effect of feed efficiency classification on SCFA production.

Parameter	High Efficiency	Neutral	Low Efficiency	*p*-Value	Difference (High − Low, 95% CI)	Hedges’ g
Total SCFA	131 ± 7.92	124 ± 5.90	123 ± 7.92	0.765	7.60 (−15.1 to 30.3)	0.36
C2:C3 ratio	1.13 ± 0.111	0.90 ± 0.082	0.90 ± 0.111	0.229	0.22 (−0.09 to 0.54)	0.57
C2 (mmol/L)	59.80 ± 2.758	52.56 ± 2.056	54.18 ± 2.758	0.119	5.61 (−2.30 to 13.5)	0.89
C3 (mmol/L)	60.07 ± 5.989	63.64 ± 4.464	61.80 ± 5.989	0.889	−1.72 (−18.9 to 15.4)	−0.09
C4 (mmol/L)	9.16 ± 0.993	7.12 ± 0.740	5.68 ± 0.993	0.057	3.47 (0.62 to 6.33)	1.05
IC5 (mmol/L)	0.87 ± 0.134	0.51 ± 0.100	0.71 ± 0.134	0.106	0.16 (−0.22 to 0.54)	0.37
C5 (mmol/L)	1.14 ± 0.089	1.14 ± 0.066	1.06 ± 0.089	0.763	0.07 (−0.17 to 0.33)	0.31
C2 (mmol %)	46.24 ± 1.625	42.75 ± 1.211	44.30 ± 1.625	0.236	1.94 (−0.72 to 6.61)	0.36
C3 (mmol %)	44.78 ± 2.480	49.93 ± 1.848	49.54 ± 2.480	0.235	−4.76 (−11.8 to 2.36)	−0.57
C4 (mmol %)	7.37 ± 1.044	5.95 ± 0.778	4.68 ± 1.044	0.203	2.69 (−0.30 to 5.69)	0.80
IC5 (mmol %)	0.69 ± 0.127	0.43 ± 0.095	0.59 ± 0.127	0.249	0.10 (−0.26 to 0.46)	0.26
C5 (mmol %)	0.88 ± 0.055	0.92 ± 0.041	0.86 ± 0.055	0.669	0.01 (−0.13 to 0.17)	0.11

*n* = 10 (high efficiency), 18 (neutral), 10 (low efficiency). Values expressed as LSMeans ± SEM adjusted for chamber measurement period. SCFA—short chain fatty acids, C2—acetate, C3—propionate, C4—butyrate, IC5—isovalerate, C5—valerate. *p*-values refer to the overall effect of feed efficiency classification. The estimated difference between high- and low-efficiency animals (high − low) is presented with its 95% confidence interval (95% CI), and Hedges’ g is reported as the standardised effect size for this planned comparison.

**Table 5:** Effect of feed efficiency classification on methane, carbon dioxide, nitrous oxide and ammonia emissions.

Parameter	High Efficiency	Neutral	Low Efficiency	*p*-Value	Difference (High − Low, 95% CI)	Hedges’ g
	Method: Gas Chromatography (Shimadzu GC-2010)					
CH_4_ (g/d)	3.23 ± 0.733	4.24 ± 0.546	2.94 ± 0.733	0.308	0.29 (−1.81 to 2.39)	0.21
CH_4_ (g/d/DMI)	3.73 ± 0.755	4.56 ± 0.563	3.05 ± 0.755	0.300	0.68 (−1.93 to 3.29)	0.48
CH_4_ (g/d/BW^0.75^)	0.18 ± 0.040	0.23 ± 0.029	0.16 ± 0.040	0.330	0.01 (−0.09 to 0.13)	0.25
	Method: Cavity ring-down spectroscopy (Picarro G2508)					
CH_4_ (g/d)	4.99 ± 0.837	6.00 ± 0.613	5.41 ± 0.823	0.599	−0.41 (−2.78 to 1.94)	−0.18
CH_4_ (g/d/DMI)	5.09 ± 0.743	5.67 ± 0.554	5.72 ± 0.743	0.792	−0.62 (−2.75 to 1.51)	−0.29
CH_4_ (g/d/BW^0.75^)	0.28 ± 0.047	0.33 ± 0.033	0.30 ± 0.045	0.732	−0.01 (−0.14 to 0.11)	−0.11
CH_4_: CO_2_ ratio	2.64 ± 0.307	2.63 ± 0.228	2.82 ± 0.307	0.875	−0.17 (−1.06 to 0.70)	−0.16
N_2_O (mg/d)	41.9 ± 9.301	52.30 ± 6.933	26.27 ± 9.301	0.094	15.6 (−11.03 to 42.38)	0.74
N_2_O (mg/d/BW^0.75^)	2.41 ± 0.540	2.96 ± 0.402	1.49 ± 0.540	0.108	0.92 (−0.62 to 2.47)	0.76
NH_3_ (g/d)	1.90 ± 0.351	2.20 ± 0.261	1.22 ± 0.351	0.097	0.67 (−0.33 to 1.68)	0.80
NH_3_ (g/d/DMI)	1.96 ± 0.416	2.22 ± 0.310	1.37 ± 0.416	0.276	0.58 (−0.60 to 1.78)	0.55
NH_3_ (g/d/BW^0.75^)	0.10 ± 0.020	0.12 ± 0.015	0.06 ± 0.020	0.116	0.04 (−0.01 to 0.09)	0.81

*n* = 10 (high efficiency), 18 (neutral) and 10 (low efficiency). Values expressed as LSMeans ± SEM adjusted for chamber measurement period. DMI—dry matter intake, BW^0.75^—metabolic body weight. d—day. *p*-values refer to the overall effect of feed efficiency classification. The estimated difference between high- and low-efficiency animals (high − low) is presented with its 95% confidence interval (95% CI), and Hedges’ g is reported as the standardised effect size for this planned comparison.

## Data Availability

Supporting data are presented throughout the article; the datasets generated during the current study are available from the corresponding author on reasonable request.

## Abbreviations

The following abbreviations are used in this manuscript:

ADG	Average daily gain
ADF	Acid detergent fibre
AIC	Akaike Information Criterion
AUC	Area under the concentration–time curve
BIC	Bayesian Information Criterion
BW	Body weight
BW^0.75^	Metabolic body weight
C2	Acetate/acetic acid
C3	Propionate/propionic acid
C4	Butyrate/butyric acid
C5	Valerate/valeric acid
CH_4_	Methane
CO_2_	Carbon dioxide
CP	Crude protein
CPI	Crude protein intake
CRDS	Cavity ring-down spectroscopy
DCP	Digestible crude protein
DDM	Digestible dry matter
DDMI	Digestible dry matter intake
DM	Dry matter
DMI	Dry matter intake
DMIe	Expected dry matter intake
DNDF	Digestible neutral detergent fibre
DOM	Digestible organic matter
EE	Ether extract
GHG	Greenhouse gas
HPLC	High-performance liquid chromatography
IC5	Isovalerate/isovaleric acid
MM	Mineral matter
MN	Microbial nitrogen
MP	Microbial protein
N	Nitrogen
NDF	Neutral detergent fibre
NDFI	Neutral detergent fibre intake
NH_3_	Ammonia
N_2_O	Nitrous oxide
OM	Organic matter
PC1	First principal component
PC2	Second principal component
PCA	Principal component analysis
PD	Purine derivatives
PDA	Absorbed purine derivatives
PDIFF	Pairwise differences
PTFE	Polytetrafluoroethylene
R^2^	Coefficient of determination
REML	Restricted maximum likelihood
RFI	Residual feed intake
RG	Residual gain
RIG	Residual intake and gain
SCFA	Short-chain fatty acids
SD	Standard deviation
SEM	Standard error of the mean
STP	Standard temperature and pressure
TMR	Total mixed ration

## Appendix A

**Table A1:** Individual animal performance traits and classification into high-, neutral-, and low-efficiency groups based on residual feed intake (RFI) and residual intake and gain (RIG).

Animal ID	BW^0.75^ (kg)	DMI (kg)	ADG (kg Day^−1^)	RFI	RG	RIG	Classification
3030	15.55	1.133	0.217	0.7059	−0.0315	−0.7374	low efficiency
3078	17.29	1.163	0.194	0.1244	−0.0232	−0.1476	neutral
3079	15.44	1.207	0.255	0.2429	−0.0018	−0.2447	low efficiency
3080	15.88	0.9945	0.301	−0.3423	0.0404	0.3827	high efficiency
3081	18.53	1.382	0.175	0.2739	−0.0378	−0.3117	low efficiency
3082	16.62	1.174	0.260	−0.1458	0.0052	0.1510	neutral
3083	16.22	1.006	0.269	0.1055	0.0210	−0.0845	neutral
3085	16.77	1.116	0.234	−0.1981	−0.0226	0.1755	neutral
3086	14.64	0.972	0.227	−0.3181	−0.0221	0.2960	high efficiency
3087	14.92	1.044	0.284	−0.1141	0.0374	0.1515	neutral
3088	14.99	1.011	0.145	−0.0577	−0.0691	−0.0114	neutral
3090	16.87	1.342	0.256	−0.1095	0.0006	0.1101	neutral
3091	13.95	1.068	0.203	0.0866	−0.0197	−0.1063	neutral
3092	14.94	1.303	0.297	0.6060	0.0634	−0.5426	low efficiency
3094	15.65	1.411	0.202	−0.0675	−0.0255	0.0420	neutral
3095	15.82	1.002	0.254	−0.1345	0.0091	0.1436	neutral
3096	17.30	1.256	0.285	−0.5780	0.0146	0.5926	high efficiency
3098	15.24	1.15	0.250	1.9538	0.0150	−1.9388	low efficiency
3099	13.57	0.874	0.226	−0.0318	−0.0121	0.0197	neutral
3100	17.12	1.269	0.271	−0.1428	0.0341	0.1769	neutral
3103	16.21	1.291	0.241	0.3061	0.0029	−0.3032	low efficiency
3105	15.64	1.191	0.224	0.2475	−0.0027	−0.2502	low efficiency
3106	15.47	1.041	0.242	0.2329	0.0435	−0.1894	low efficiency
3107	14.85	1.095	0.250	−0.4454	0.0030	0.4484	high efficiency
3108	16.08	0.98	0.276	−0.2427	0.0060	0.2487	high efficiency
3109	18.18	1.428	0.250	0.0194	0.0175	−0.0019	neutral
3110	16.58	1.331	0.257	−0.0730	−0.0079	0.0651	neutral
3112	16.22	0.88	0.255	−0.4848	0.0216	0.5064	high efficiency
3113	13.10	0.937	0.276	−0.1700	0.0217	0.1917	neutral
3114	15.89	1.157	0.288	−0.3606	0.0239	0.3845	high efficiency
3115	15.98	1.242	0.265	−0.5153	0.0512	0.5665	high efficiency
3116	15.54	0.881	0.246	−0.7335	0.0092	0.7427	high efficiency
3120	15.09	1.015	0.220	−0.3157	−0.0811	0.2346	high efficiency
3122	16.13	1.378	0.228	−0.0151	−0.0019	0.0132	neutral
3123	15.43	1.033	0.204	−0.1144	−0.0889	0.0255	neutral
3124	14.77	1.003	0.301	−0.1174	0.0592	0.1766	neutral
3125	13.39	0.831	0.197	0.5307	−0.0424	−0.5731	low efficiency
3126	19.18	1.424	0.288	0.3890	0.0482	−0.3408	low efficiency

BW^0.75^—metabolic body weight, DMI—dry matter intake, ADG—average daily gain, RFI—residual feed intake, RG—residual gain, RIG—residual intake and gain.
